# Decoding Naturalistic Episodic Memory with Artificial Intelligence and Brain‐Machine Interface

**DOI:** 10.1002/advs.202520125

**Published:** 2026-02-28

**Authors:** Dong Song

**Affiliations:** ^1^ Department of Neurological Surgery Department of Biomedical Engineering Neuroscience Graduate Program Neurorestoration Center University of Southern California Los Angeles California USA

**Keywords:** artificial intelligence, brain‐machine interface, episodic memory, hippocampus, multimodal modeling

## Abstract

Episodic memory integrates what, where, and when of experience into a coherent autobiographical narrative. Decades of research have identified hippocampal place, time, and concept cells as neural correlates of these components. Yet a major challenge remains: real‐life memory encoding occurs in high‐dimensional, naturalistic settings, where multimodal sensory, emotional, and cognitive processes intertwine across time and context. Traditional paradigms and analytical tools are insufficient to decode the neural activity underlying such complex experiences. Recent advances in artificial intelligence (AI) offer new means to address this challenge. AI models, such as variational autoencoders and multimodal alignment frameworks, can extract latent representations from neural and behavioral data, capturing the naturalistic structure of memory encoding. Large language models further provide powerful frameworks for interpreting subjective memory reports, linking verbal narratives to memory encoding. When integrated with closed‐loop brain–machine interfaces (BMIs) capable of recording from and manipulating large populations of neurons in relevant brain regions, these tools make it possible to address the long‐standing questions: how to decode memory codes during naturalistic behaviors and whether these memory codes causally generate memories rather than merely correlate with them. This integrated AI–BMI framework outlines a roadmap from mapping to engineering memory, with implications for Alzheimer's disease, traumatic brain injury, and PTSD.

## Graphical abstract

Episodic memory weaves together what, where, and when of experience into a personal narrative. Cutting‐edge AI models may decode this intricate process in real‐life settings, revealing how neural activity encodes naturalistic memories. By merging AI with brain–machine interfaces, researchers are edging closer to mapping and even engineering memory, thereby reshaping our understanding of cognition and potentially curing neurological diseases.

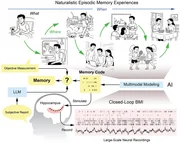

Memory encoding is the process by which information from our experiences is transformed into neural activity patterns that can later be stored and retrieved. In episodic memory, encoding binds together what, where, and when of events into a coherent autobiographical narrative [1].

For example, if you ask me what I did yesterday, I might recall: waking up at 6:30 a.m. to my phone alarm, taking the Metro to the USC campus in the morning. On my way to the lab, I noticed a squirrel darting across the path. In the morning, I met with my students in the lab, and in the afternoon, I gave a lecture on neural signal processing in the classroom. After work, I played tennis with my wife in a local park and had dinner in our dining room in the evening (Figure 1, top). Episodic memory enables this type of autobiographical reconstruction of personal experience, linking distinct events involving different objects (phone, car, train, squirrel, students, tennis racquets, and dinner table), occurring in different places (bedroom, train station, campus, lab, park, and dining room), and unfolding across different times (morning, afternoon, and evening).

**FIGURE 1:**
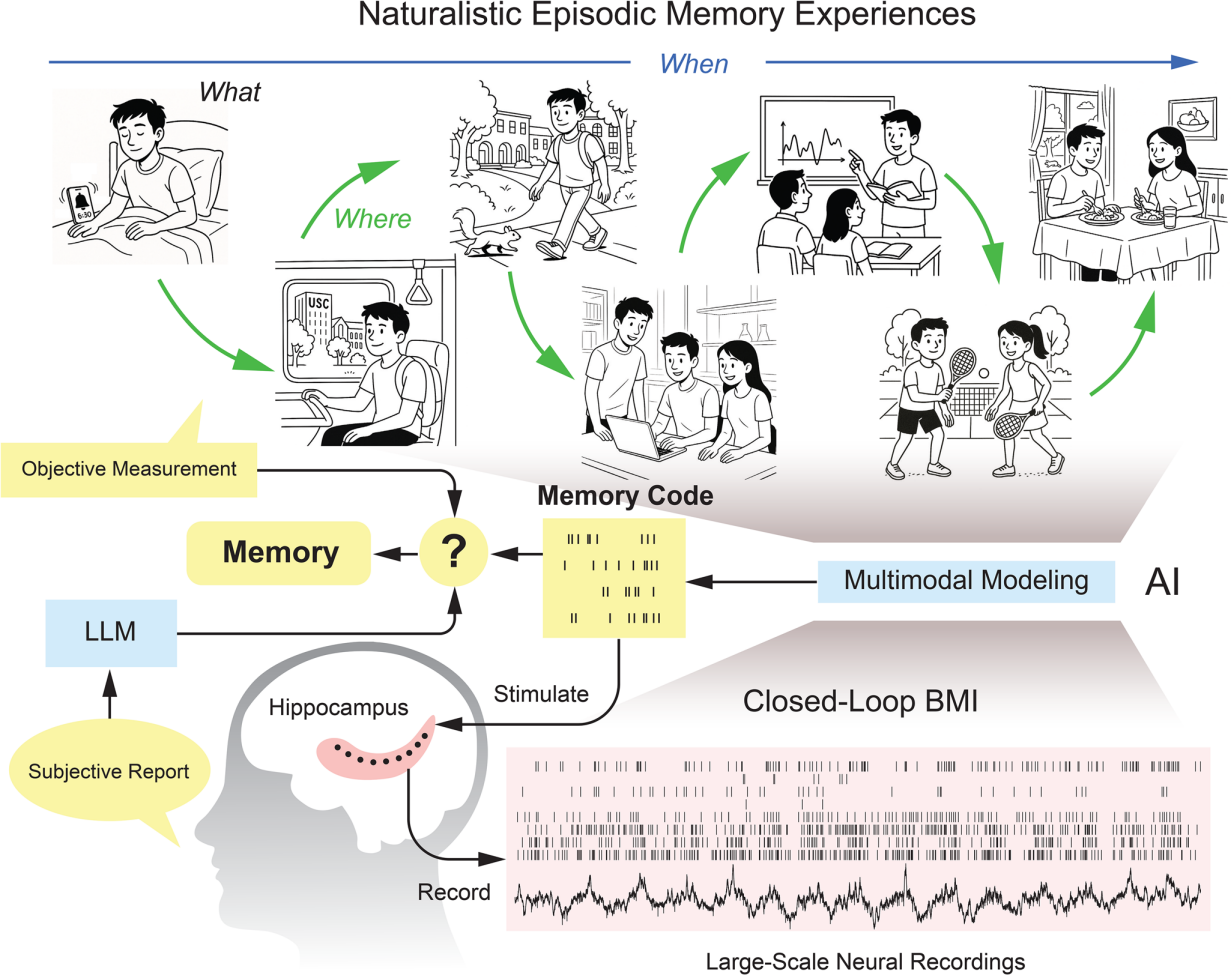
Integrating AI and closed‐loop brain–machine interfaces (BMIs) to decode and test naturalistic episodic memory. Top: Naturalistic episodic memories integrate information about *what*, *where*, and *when* into rich, location‐dependent experiences that unfold across time. Middle: A multimodal AI modeling framework can align latent dynamics derived from large‐scale neural recordings with those extracted from behavioral and experiential data, enabling the decoding of complex memory representations under naturalistic conditions. Bottom: A closed‐loop BMI paradigm can then be used to causally test these models by perturbing or reinstating specific memory codes during memory encoding and retrieval. Subjective reports and objective measurements of behavioral performance together provide validation of the reconstructed or modified memory experience.

The foundational case of patient H.M. revealed that lesions to medial temporal lobe structures, including the hippocampus, the entorhinal cortex, the amygdala, and adjacent cortical regions, abolish the ability to form new episodic memories while sparing many other cognitive functions [2]. This landmark finding provided the first compelling evidence that episodic memory depends on the integrity of the medial temporal lobe memory system. Subsequent investigations, including studies of patient R.B. and selective lesion experiments in nonhuman primates with damage confined to the hippocampus [3, 4, 5], further narrowed this system and established the hippocampus as a central hub for episodic memory. These discoveries catalyzed decades of electrophysiological and neuroimaging research aimed at understanding how the hippocampus encodes, binds, organizes, and stores the elements of personal experience. Since then, the study of episodic memory encoding has primarily focused on identifying the neural correlates of memory components within the hippocampus and its interconnected cortical regions, with the goal of understanding how distributed neural activity gives rise to coherent, retrievable episodes.

## 1 From Space and Time to Unified Episodic Memory Codes

In rodents, the first major discovery of the neural correlates of memory components came with the identification of place cells, hippocampal neurons that fire when the animal occupies a specific location [6]. Grid cells were later discovered in the entorhinal cortex, forming a metric for spatial navigation [7]. These findings reinforced a view of the hippocampus as a spatial map [8].

Temporal coding emerged with the later discovery of time cells, which fire in a sequential order to bridge temporal gaps between events, providing a neural representation of elapsed time and supporting the organization of events into temporal sequences [9]. Importantly, these spatial and temporal codes are not independent of other features; hippocampal neurons can conjunctively encode spatial location, temporal order, and object identity [9, 10, 11, 12, 13], and in some cases act as human episode cells that represent unique, integrated events [14]. These findings are consistent with a relational memory framework in which the hippocampus binds the “what,” “where,” and “when” of experience into structured episodic representations. This reframed the hippocampus as a relational memory system, unifying disparate elements of experience [12]. By bridging human memory research and rodent spatial studies, these studies resolve an early disconnect: in humans, hippocampal function was primarily linked to episodic memory, whereas in rodents it was initially framed almost exclusively in spatial terms.

While when, where, and what are all essential components of episodic memory, they differ fundamentally in representational complexity. Time is inherently 1D, unfolding along a linear axis. Space is typically 2D for most terrestrial animals, but becomes 3D for species navigating volumetric environments, such as birds and bats [15]. Objects, however, occupy an extremely high‐dimensional representational space encompassing shape, color, texture, function, semantic associations, and affective valence. Exhaustively encoding all possible objects is neither biologically plausible nor computationally efficient. A likely neural strategy is to reduce this complexity by encoding abstract and salient features of objects, thereby compressing the high‐dimensional space into a more tractable representational form.

Early nonhuman primate studies suggest that objects are represented as categories in the hippocampus [16]. Subsequent recordings from the human medial temporal lobe revealed concept‐selective neurons that respond invariantly to specific individuals or abstract concepts, independent of visual details or presentation modality [17]. In humans, researchers have also identified category cells similar to those observed in animals [18, 19, 20, 21] as well as more specific object cells [22, 23]. Concept, category, and object cells are all high‐level “what” cells that encode what an object is rather than its low‐level sensory properties, and all three exhibit selectivity, responding more strongly to some objects than to others. Although these terms are sometimes used interchangeably, important distinctions exist: concept cells encode abstract concepts that generalize across many specific instances and even across sensory modalities (for example, different pictures and the written or spoken name of the same person), whereas category cells encode membership in broader classes (such as “faces” or “animals”) and respond to many different members of a category without distinguishing individuals. Object cells, in contrast, encode particular objects or narrow sets of highly similar objects, typically within a single modality (often vision), and are more sensitive to specific views or features.

Early work on “what” cells focused on rate coding at the level of single neurons, defining a cell type as one that increases its firing rate within a specific time window when a particular category, concept, or object is encountered. More recently, researchers have begun to examine how objects are encoded in the spatio‐temporal firing patterns of hippocampal neuronal populations [24]. Decoding memory from hippocampal spiking activity was framed as a classification problem, with spatio‐temporal spike patterns as inputs and visual image categories as outputs [25]. Results showed that visual image categories can be reliably decoded from hippocampal population spiking activity, and individual categories are represented with a distributed temporal code. These findings collectively indicate that hippocampal object representations are dynamic, temporally structured, and organized across multiple levels of abstraction, supporting both flexible generalization and efficient retrieval of episodic memories.

In addition to single‐unit spiking activity, population‐level field signals, such as local field potentials (LFP), intracranial EEG (iEEG), electrocorticography (ECoG), and even scalp EEG, also carry rich information about memory processes. Oscillatory dynamics across multiple frequency bands, including theta rhythms [26, 27, 28], gamma oscillations [26, 29], and sharp‐wave ripples [30], have each been linked to distinct aspects of memory encoding, consolidation, and retrieval. These rhythms reflect coordinated population activity and provide more accessible and stable biomarkers of underlying hippocampal–cortical network states. Leveraging this knowledge, closed‐loop Brain‐Machine Interface (BMI) systems have begun to use these oscillatory features as real‐time biomarkers to guide targeted neuromodulation aimed at enhancing or perturbing memory processes [31, 32, 33, 34, 35, 36].

## 2 AI as a New Tool for Decoding Naturalistic Episodic Memory

Controlled laboratory paradigms, such as delayed match‐to‐sample [10], paired‐associate learning [5], virtual navigation [37], and object‐location tasks [38], have been invaluable for isolating specific variables and dissecting mechanisms of memory encoding. However, these paradigms lack the richness and multidimensionality of natural episodes during naturalistic behaviors.

Naturalistic behaviors refer to actions and experiences that unfold in environments approximating the complexity and variability of everyday life. They integrate not only when, where, and what, but also multimodal sensory inputs, ongoing movement, spontaneous attention shifts, social or emotional context, and continuous interactions with the environment—in contrast to traditional laboratory tasks that isolate single variables and tightly constrain behavior.

Recent human studies have begun to bridge this gap by adopting multimodal recording approaches that align behavior with neural activity in increasingly naturalistic settings. These paradigms span a wide spectrum, including allowing patients with implanted electrodes to move freely within the ICU rather than remain confined to bed [39], employing virtual reality–based navigation tasks [40, 41], recording neural activity during repeated story listening [42] or movie watching [43, 44], utilizing game‐based or immersive behavioral tasks [45], and enabling subjects to navigate environments that more closely approximate real‐world conditions [46, 47, 48, 49]. In this perspective, I consider any methodological advance that integrates multimodal neural and behavioral recordings in less constrained subjects as a meaningful step toward greater behavioral naturalism.

Despite such advances, simultaneous recording of complex behaviors and large‐scale neuronal spiking activity, the temporal granularity essential for decoding episodic memory, remains technically challenging. Moreover, naturalistic tasks introduce profound analytical complexity. In real life, stimuli are uncontrolled, modalities are intertwined, and multiple objects and contexts overlap across time and space. This results in high‐dimensional representations, where neural activity varies not only with multimodal sensory inputs but also with fluctuating cognitive and emotional states. Decoding these neural dynamics, therefore, requires new analytical frameworks capable of linking high‐dimensional, dynamical neural recordings to equally complex behavioral data. Such methods must integrate multimodal information and adapt to the temporal and contextual fluidity that defines real‐life naturalistic episodic memory.

Modern artificial intelligence (AI) provides powerful tools for modeling the complex, high‐dimensional dynamics of episodic memory. Deep learning architectures are well‐suited to extract meaningful structure from large and heterogeneous datasets that combine neural and behavioral features [50]. Moreover, advances in interpretable and mechanistically informed deep learning frameworks enable these models not only to decode neural activity but also to illuminate the structure of neural representations and coding principles [51]. For example, convolutional neural networks can extract object information from videos [52]; variational autoencoders (VAEs) can reduce the dimensionality of complex neural and behavioral data, uncovering latent variables that capture the essential features of memory representations [53, 54]. To model temporal dynamics, recurrent neural networks (RNNs) and transformer architectures excel at capturing temporal associations that span seconds to minutes [55, 56], matching the timescales of memory encoding and retrieval. In neurobiological studies, where extensive labeled training data are often limited, contrastive learning and other self‐supervised frameworks can be leveraged to build effective decoding models using weak or indirect labels, mitigating the need for exhaustive annotation [57, 58, 59].

A particularly promising approach is multimodal modeling, which focuses on integrating and explaining complex relations across different data modalities [60, 61]. Using this approach, neural activity and behavioral experience during naturalistic settings can be more effectively extracted and further jointly embedded into a shared latent space (Figure 1, middle). For instance, separate autoencoders may be trained in parallel to extract latent representations from neural activities, such as spikes and LFPs, and from multimodal behavioral streams, such as visual scenes, sounds, and semantic contents, recorded during the same episodes. By aligning these latent spaces, researchers can uncover correspondences between internal neural trajectories and the external structure of complex events, without relying on predefined semantic categories or restrictions to lower‐dimensional behavioral variables, as in the more constrained laboratory settings [62]. This data‐driven alignment enables the model to discover naturalistic memory categories directly from experience, capturing the multidimensional richness of real‐world episodic memories. Such approaches have the potential not only to reveal previously unrecognized structures in memory encoding but also to support more ecologically valid, closed‐loop manipulations in which stimulation parameters are guided by latent neural‐behavioral dynamics.

Beyond methodology, AI‐driven modeling may also catalyze a conceptual shift in how memory is studied, altering the balance between reductionist and inductionist approaches and expanding the scope of inquiry from isolated mechanisms to emergent systems‐level principles [63]. Traditional reductionism isolates individual variables, such as specific cell types, firing patterns, or stimulus‐response mappings, to build mechanistic explanations from the ground up. This framework produced foundational insights into spatial coding, temporal sequences, and categorical object representation. However, with the advent of large‐scale, high‐density recordings, immersive naturalistic paradigms, and powerful AI‐based analysis, it is now possible to complement this tradition with an inductionist perspective—deriving principles directly from the full complexity of unconstrained, multimodal data. Inductionist modeling has the potential to reveal emergent properties of episodic memory that are difficult to anticipate from component‐level analyses, such as cross‐modal generalization (e.g., recalling a scene from its soundtrack alone) or modality convergence (e.g., linking a voice to a face and a place without direct co‐occurrence). Integrating these two perspectives may lead to a unified understanding of how diverse experiential elements coalesce into coherent, retrievable episodes.

## 3 Engineering Causal Tests with Closed‐Loop Brain‐Machine Interface

Most early studies of episodic memory have been correlational in nature. Neural recordings have revealed strong associations between specific patterns of brain activity and successful memory formation, leading to the inference that these regions are critical for episodic memory and its encoding mechanisms. However, correlation alone does not imply causation [64]. Observing a neural pattern during encoding does not prove that the pattern itself is responsible for generating the memory. To establish causality, we must perturb or manipulate these neural codes and demonstrate that such interventions directly alter memory formation or retrieval.

Rodent studies have pioneered causal approaches using optogenetics. In a landmark series of experiments, it was shown that activating hippocampal neurons tagged during exposure to a specific context could induce a fear memory when paired with an aversive stimulus [65]. While groundbreaking, these manipulations were limited to static ensembles of neurons associated with single contextual memories. It remains unclear whether inducing sequences of spatiotemporal activity patterns across multiple ensembles can recreate the richness and complexity of naturalistic episodic memories [30].

A complementary line of research arose from a specific form of the closed‐loop BMI, that is, the hippocampal memory prosthesis [66]. Using multi‐input multi‐output (MIMO) nonlinear dynamic models [67, 68], neural activity recorded from hippocampal CA3 was transformed into predicted CA1 outputs during successful encoding. Delivering these model‐derived electrical stimulation patterns to CA1 enhanced recall in rodents [69], nonhuman primates [70], and human epilepsy patients [71], demonstrating that artificially generated neural codes can causally influence memory formation. However, current implementations are constrained by technical limitations, including small‐scale recordings and stimulation, imperfectly controlled effects of direct electrical stimulation via microelectrodes [72, 73], restricted behavioral tasks, and simplified memory variables. In addition, the MIMO model generates stimulation patterns based on upstream CA3 activity by modeling CA3–CA1 circuit input–output transformations, rather than deriving stimulation patterns directly from decoded memory representations [24, 74]. Whether writing high‐dimensional, temporally structured memory code into the hippocampus can evoke genuine episodic experiences remains an open and fundamental question.

Looking ahead, we envision a combined AI and closed‐loop BMI framework for identifying and testing the causal neural codes underlying episodic memory (Figure 1, bottom). A BMI is defined as a system that records brain activity and translates it into control signals for external devices. In this context, a closed‐loop memory BMI records neural activity from memory‐related circuits, decodes patterns linked to successful encoding or retrieval (memory code), and delivers targeted feedback, such as electrical stimulation or behavioral modulation, to enhance or modify memory [30, 36, 75, 76, 77, 78, 79, 80, 81]. Unlike general neural prostheses, a memory BMI must operate with millisecond‐scale temporal precision, adapting to ongoing cognitive states. The MIMO‐based hippocampal prosthesis exemplifies this approach by “writing” model‐derived memory codes back into the brain, restoring and enhancing memory functions in individuals with memory impairment.

Within this framework, multimodal modeling serves as the foundation for mapping relationships between neural activity and experiential variables, characterizing hippocampal representations of episodic events, such as space, time, and objects, as distributed spatio‐temporal spike patterns across interacting regions. Closed‐loop BMI systems combining large‐scale recording with precise stimulation could then manipulate these patterns in real time [82, 83]. Behavioral outcomes and subjective reports would determine whether such manipulations generate, strengthen, or disrupt specific episodic experiences, thereby distinguishing truly causal neural codes from merely correlational signatures.

Although significant challenges remain, including developing AI models that are not only accurate but also interpretable [84], robust to limited training data [85], and capable of quantifying uncertainty in high‐dimensional neural–behavioral mappings [86, 87], as well as overcoming technical hurdles in closed‐loop BMI experiments [88], uniting AI‐driven decoding with closed‐loop neural intervention offers a path toward definitively identifying and causally validating the neural codes underlying episodic memory.

## 4 Why Human Memory Matters: The Subjective Dimension

Episodic memory is defined not only by its informational content but also by its subjective phenomenology—Tulving's notion of “mental time travel [89].” While animal models enable us to infer episodic‐like memory behaviorally, they offer no insight into the inner, phenomenological experience of remembering. Human research, by contrast, uniquely enables the integration of first‐person reports with neural recordings, allowing us to directly probe memory quality.

An early and striking example of memory elicitation through targeted neural intervention was reported in a study where deep‐brain stimulation (DBS) of the hypothalamus–fornix pathway unexpectedly triggered vivid autobiographical memories in a patient undergoing treatment for obesity [90]. These déjà vu‐like experiences were reproducible under double‐blind conditions, and stimulation was found to enhance recollection without affecting familiarity‐based recognition. Such subjective phenomena have been systematically examined using structured assessments of memory phenomenology, including the Vividness of Visual Imagery Questionnaire (VVIQ) [91], the Autobiographical Memory Interview (AMI) [92], and trial‐by‐trial ratings of memory richness or confidence [93].

These measures can be enhanced with large language models (LLMs) [55], which enable automated, fine‐grained scoring of free‐text responses according to established rubrics (Figure 1, bottom‐left). LLMs can analyze participants’ narratives to extract features such as vividness, emotional tone, temporal detail, and sensory richness, yielding multidimensional profiles that surpass traditional manual ratings. With a rigorous calibration framework that involves cross‐validation against human experts, they may support standardized administration to reduce interviewer bias and enhance reliability across studies [94]. When combined with neural and behavioral data, LLM‐derived metrics have the potential to provide dynamic, high‐resolution assessments of subjective memory experience. Ultimately, LLM‐assisted instruments may help quantify the qualitative and experiential dimensions of recall, extending evaluation beyond objective measurement accuracy alone. Integrating these enhanced measures with targeted neural stimulation offers a rigorous framework for testing whether AI‐guided and closed‐loop BMI‐based interventions influence not only what is remembered, but also how it is consciously experienced.

## 5 Looking Ahead: From Mapping to Engineering Memory

The convergence of AI‐based decoding, closed‐loop BMIs, and cross‐species neuroscience offers an exciting trajectory from merely observing neural correlates to engineering memory processes themselves. This roadmap begins with identifying neural codes under controlled experimental paradigms, extends to decoding these codes in naturalistic behavioral contexts, advances to real‐time manipulation of neural activity through adaptive stimulation, and ultimately culminates in clinical translation, where these insights may be applied to restore or enhance memory function in patients. Potential clinical applications span Alzheimer's disease, traumatic brain injury, and post‐traumatic stress disorder (PTSD), conditions characterized by disrupted hippocampal–cortical encoding pathways. By integrating AI‐driven decoding with BMI‐mediated intervention, future memory prostheses could selectively reinforce weakened encoding processes or suppress maladaptive memory traces.

As the field transitions from observation to intervention, ethical considerations become paramount. Manipulating human memory raises profound questions about consent, personal identity, authenticity of recollection, equity, and potential misuse, such as memory manipulation [95, 96]. Safeguarding these frontiers will require not only technical precision but also robust ethical and regulatory frameworks, ensuring that the engineering of memory advances human well‐being while preserving autonomy and dignity [97, 98].

## Funding

This research was partially supported by the Defense Advanced Research Projects Agency (DARPA) Restoring Active Memory (RAM) program (N66001‐14‐C‐4016), partially supported by the NIH/NIDA BRAIN Initiative – Theories, Models and Methods (TMM) program (RF1DA055665/1R01EB031680), and partially supported by the DARPA Investigating how Neurological Systems Process Information in REality (INSPIRE) program (HR0011‐25‐3‐0142).

## Conflicts of Interest

The author declares no conflicts of interest.
